# Supplementary material for: IL-18 Binding Protein, a biomarker of strength maintenance after surgery but reduced physical performance in age-related sarcopenia
Source: PLoS One. 2026 Jan 27;21(1):e0340493. doi: 10.1371/journal.pone.0340493 (PMC12843554; doi:10.1371/journal.pone.0340493)
Supplement: S1 File — (DOCX) [file pone.0340493.s001.docx]

**Table S1 Demographics of the surgery cohort used for the SOMAscan**

|  | All patients |
| --- | --- |
| **Demographic data** |  |
| Age (yr) | 70 (60,75) |
| BMI (kg/m2) | 27.4±4.2 |
| EuroSCORE 2 | 2.2 (1.3-5.5) |
| **Critical care data** |  |
| ICU length of stay, days | 2 (1-4) |
| Hospital length of stay, days | 11 (7-14) |
| **Muscle data** |  |
| QMVC d0 (kg) | 27.6±7.1 |
| QMVC (% change ) | 7.9±13.0 |
| Hand grip d0 (kg) | 28.0±7.7 |
| Hand grip (% change) | 18.2±27.5 |

**Table S2 Demographics of the LACE cohort used**

|  | Women | Men |
| --- | --- | --- |
| Number | **68** | **61** |
| Age (years) | **78 (74, 84)** | **77 (74, 84)** |
| weight (kg) | **62.9 (57.5, 71.5)** | **81.1 (74.9, 89.9)** |
| height (cm) | **157 ± 7** | **171 ± 6** |
| BMI (kg/m^2^) | **26.2 (24.1, 28.3)** | **27.7 (25.6, 30.2)** |
| SARC-F | **4 (3, 5)** | **3 (3, 4)** |
| Muscle mass (kg/m^2^) | **5.74 (5.44, 5.99)** | **7.23 ± 0.64** |
| 6MWD baseline | **290 (250, 359)** | **325 (240, 399)** |
| 6MWD 12 months | **350 (285, 380)** | **328 (250, 428)** |
| SPPB baseline | **7 (5, 9)** | **7 (6, 9)** |
| SPPB 12 months | **8 (6, 9)** | **8 (6 ,9)** |
| Grip strength baseline (kg) | **13.8± 3.5** | **23.5 ± 5.8** |
| Grip baseline/arm muscle (kg/kg) | **4.0 (3.2, 4.9)** | **3.9 (3.2, 4.5)** |
| Grip strength 12 months (kg) | **15.2 ± 3.3** | **24.2 ± 6.1** |
| QMVC baseline (kg) | **9.9 (6.6, 12.1)** | **15.6 (11.1, 19.9)** |
| QMVC baseline/leg muscle (kg/kg) | **0.9 (0.6, 1.2)** | **1.0 (0.7, 1.3)** |
| QMVC 12 months (kg) | **10.3 (7.4, 12.7)** | **15.6 (12.2, 23.3)** |
| IL-18BP baseline (pg/mL) | **9.1 (6.0, 12.5)** | **7.7 (5.5, 11.7)** |
| IL-18BP 12 months (pg/mL) | **9.4 (6.5, 13.1)** | **7.4 (6.0, 13.3)** |

**Tables S3-S6 see excel sheet**

**Table S7 see excel sheet**

**Table S8: Gene Set enrichment analysis using the GO: Biological processes gene sets for muscle genes ranked by association with IL-18BP prior to surgery**

| GO:BP gene sets | NES | NOM p-val | FDR q-val |
| --- | --- | --- | --- |
| ATP SYNTHESIS COUPLED ELECTRON TRANSPORT | -2.76 | <0.001 | <0.001 |
| MITOCHONDRIAL ELECTRON TRANSPORT NADH TO UBIQUINONE | -2.52 | <0.001 | <0.001 |
| AEROBIC RESPIRATION | -2.50 | <0.001 | <0.001 |
| OXIDATIVE PHOSPHORYLATION | -2.45 | <0.001 | <0.001 |
| ELECTRON TRANSPORT CHAIN | -2.43 | <0.001 | <0.001 |
| PROTON MOTIVE FORCE DRIVEN ATP SYNTHESIS | -2.36 | <0.001 | <0.001 |
| CELLULAR RESPIRATION | -2.27 | <0.001 | 0.001 |
| ATP BIOSYNTHETIC PROCESS | -2.19 | <0.001 | 0.005 |
| MITOCHONDRIAL RESPIRATORY CHAIN COMPLEX ASSEMBLY | -2.19 | 0.000 | 0.005 |
| MITOCHONDRIAL ELECTRON TRANSPORT CYTOCHROME C TO OXYGEN | -2.15 | 0.002 | 0.008 |
| TRICARBOXYLIC ACID CYCLE | -2.13 | 0.000 | 0.010 |
| NADH DEHYDROGENASE COMPLEX ASSEMBLY | -2.09 | 0.002 | 0.015 |
| TRANSFORMING GROWTH FACTOR BETA PRODUCTION | -2.07 | 0.000 | 0.017 |
| HOMOLOGOUS RECOMBINATION | -2.01 | 0.000 | 0.031 |

**Table S9: Associations of Total and Free IL-18 and IL-37 with muscle phenotype prior to surgery**

| Protein | RFcsa | | Hand grip | | QMVC | |
| --- | --- | --- | --- | --- | --- | --- |
|  | r | p | r | p | r | p |
| Total IL-18 day 0 | -0.071 | 0.690 | -0.037 | 0.836 | -0.072 | 0.680 |
| Free IL-18 day 0 | 0.158 | 0.396 | 0.184 | 0.330 | 0236 | 0.205 |
| Total IL-37 day 0 | -0.384 | 0.015 | -0.203 | 0.205 | -0.334 | 0.034 |

RFcsa: rectus femoris cross sectional area, QMVC: Quadriceps maximal voluntary contraction, R values are Spearman’s rank correlation coefficients

**Table S10: Associations of Total and Free IL-18 and IL-37 24 hrs after surgery with change in muscle phenotype 7 days post-surgery**

| Protein | RFcsa % loss by day 7 | | Hand grip % loss by day 7 | | QMVC % loss by day 7 | |
| --- | --- | --- | --- | --- | --- | --- |
|  | r | p | r | p | r | p |
| Total IL-18 day 0 | -0.023 | 0.896 | -0.077 | 0.666 | -0.010 | 0.956 |
| Free IL-18 day 0 | 0.018 | 0.918 | 0.079 | 0.666 | -0.155 | 0.403 |
| Total IL-37 day 0 | 0.252 | 0.146 | 0.062 | 0.726 | -0.133 | 0.460 |

RFcsa: rectus femoris cross sectional area, QMVC: Quadriceps maximal voluntary contraction, R values are Spearman’s rank correlation coefficients
