# Supplementary material for: IL-18 Binding Protein, a biomarker of strength maintenance after surgery but reduced physical performance in age-related sarcopenia
Source: PLoS One. 2026 Jan 27;21(1):e0340493. doi: 10.1371/journal.pone.0340493 (PMC12843554; doi:10.1371/journal.pone.0340493)
Supplement: S4 File — (ZIP) [file pone.0340493.s004.zip › Supplementary figure Legends.docx]

**Figure S1. Volcano plot showing differential plasma levels of proteins in response to aortic surgery.**

Plasma levels of 1300 proteins were quantified by SOMAscan in samples taken from male patients before (day 0) and 24h after aortic surgery (day 1). Data were log transformed in metaboanalyst 5.0 and differential levels were determined by ANOVA. The data are presented as log_2_Fold change between day 0 and day 1 plotted against log_10_ P value (prior to correction for multiple testing).

**Figure S2. ELISA measurements of IL-18BP show reasonable agreement with SOMAscan measurements of the same protein.**

Levels of IL-18BP determined by SOMAscan and ELISA measured in duplicate samples from the same patient were compared. The measurements showed reasonable agreement (A) and demonstrated a marked increase in IL-18BP following surgery (B).

**Figure S3. ELISA measurements of IL-18 and IL-37 in patients in the acute sarcopenia cohort**

Levels of IL-18 and IL-37 were measured in patients before and 24h after surgery by ELISA. IL-18 levels did not change in response to surgery (A) but there was a marked increase in circulating levels of IL-37 (p<0.001, B)
