# Supplementary figures and images for: IL-18 Binding Protein, a biomarker of strength maintenance after surgery but reduced physical performance in age-related sarcopenia
Source: PLoS One. 2026 Jan 27;21(1):e0340493. doi: 10.1371/journal.pone.0340493 (PMC12843554; doi:10.1371/journal.pone.0340493)

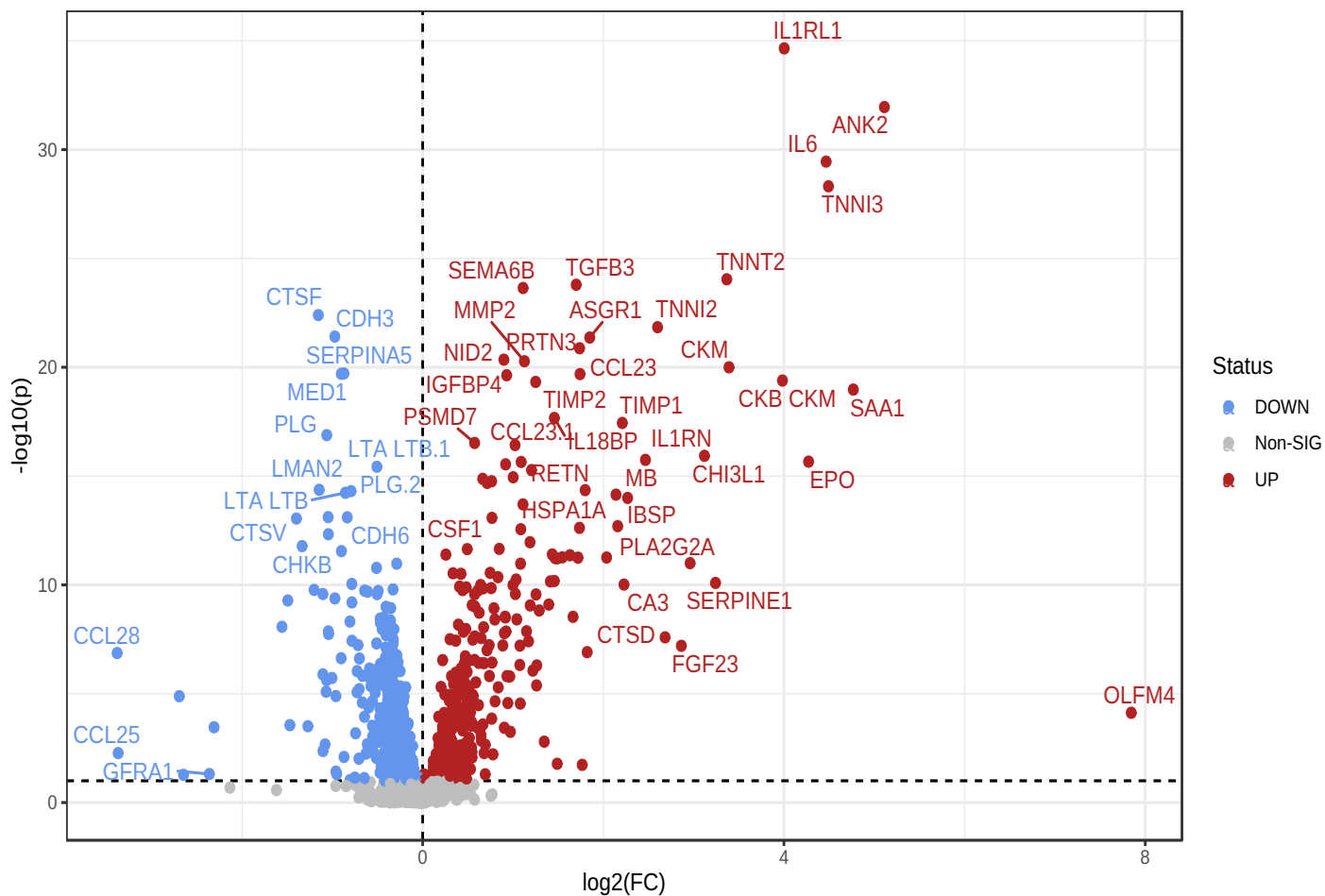

Figure S1

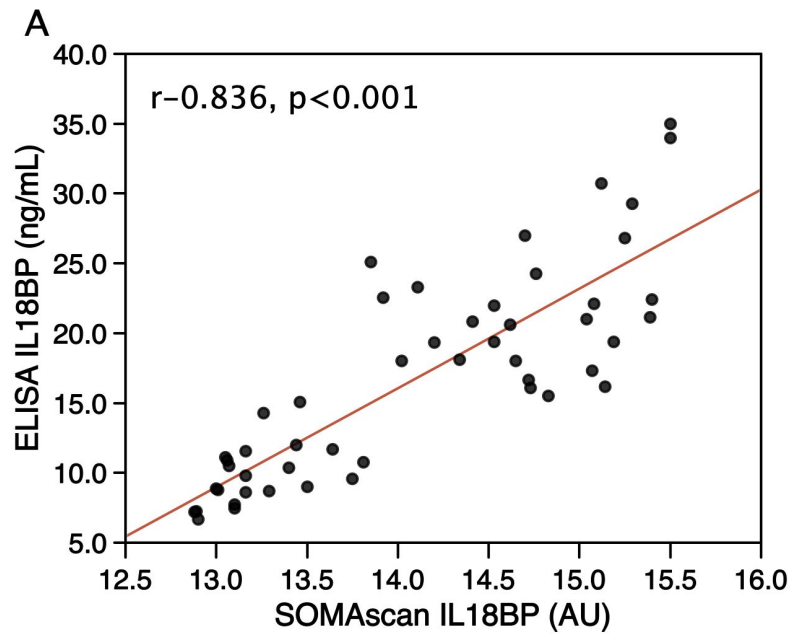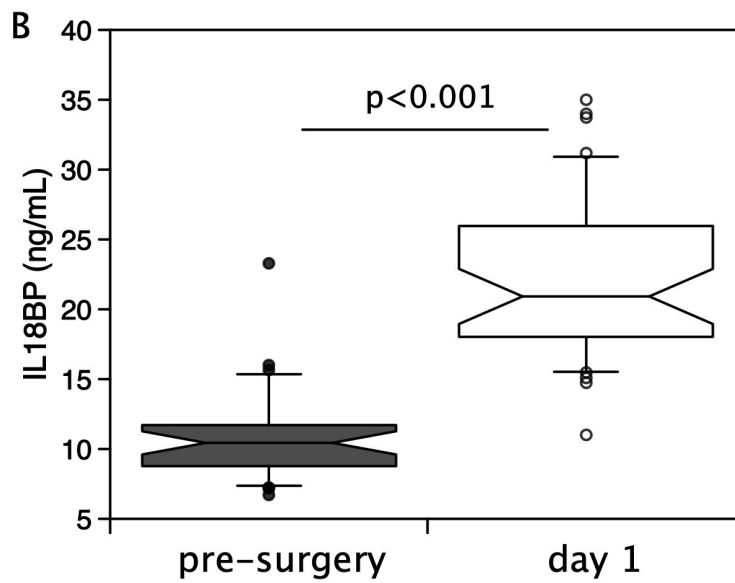

Figure S2

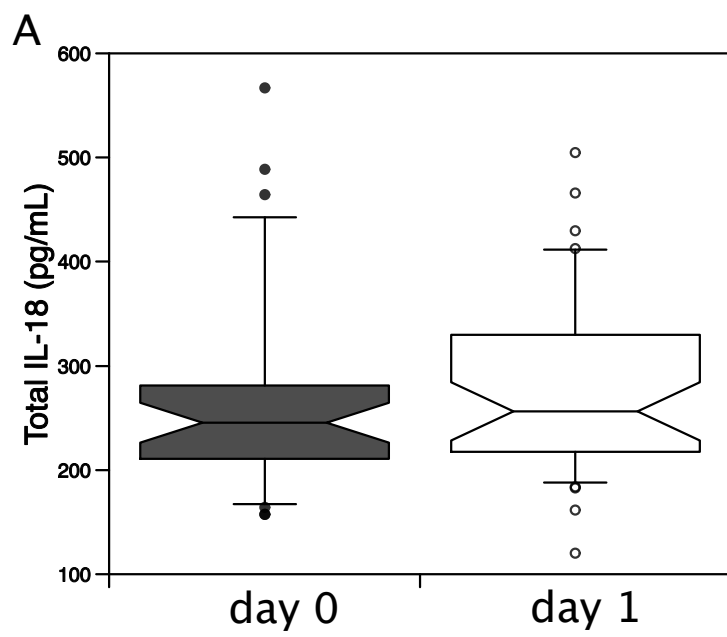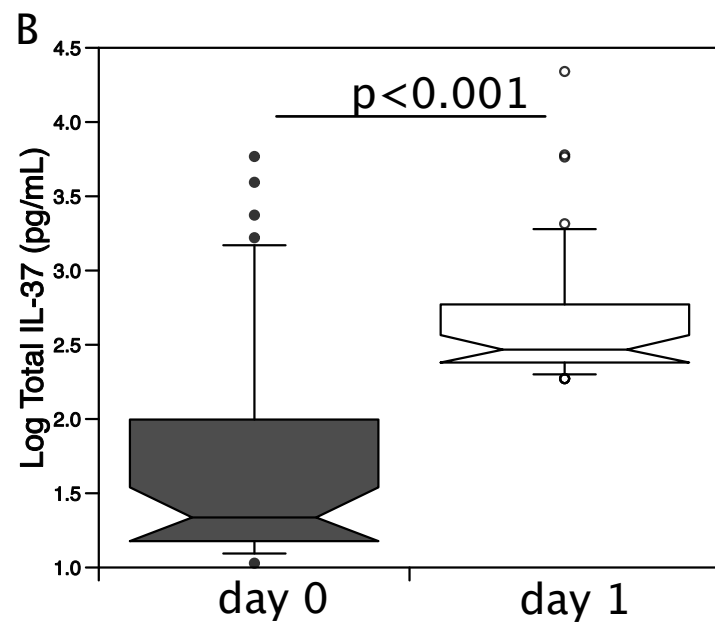

Figure S3

Supplement: S4 File — (ZIP) [file pone.0340493.s004.zip › Supplementary Figures S1-S3.pdf]
