# Supplementary material for: IL-18 Binding Protein, a biomarker of strength maintenance after surgery but reduced physical performance in age-related sarcopenia
Source: PLoS One. 2026 Jan 27;21(1):e0340493. doi: 10.1371/journal.pone.0340493 (PMC12843554; doi:10.1371/journal.pone.0340493)
Supplement: Attachment — Submitted filename: Response IL18BP letter.docx [file pone.0340493.s005.docx]

Dear Dr Thiyagarajan,

Thank you for the chance to respond to your and the referees’ comments on our paper. We thank you and them for the time that they dedicated to reviewing our work and suggesting the changes and clarifications that they have done. We feel that this has improved the quality of the paper. We hope that they are satisfied with the modifications that we have made and accept the explanation of our rationale for not changing things where we have not.

We have presented them in the document using the comments (in blue) as a framework and have included our responses and the modifications in black text.

With these improvements we hope that the paper is now acceptable to you for publication

Yours Sincerely

Paul Kemp

**Journal Requirements:**

1. When submitting your revision, we need you to address these additional requirements.

Please ensure that your manuscript meets PLOS ONE's style requirements, including those for file naming. The PLOS ONE style templates can be found at

https://journals.plos.org/plosone/s/file?id=wjVg/PLOSOne formatting sample main body.pdf and

https://journals.plos.org/plosone/s/file?id=ba62/PLOSOne formatting sample title authors affiliations.pdf

2. We note that there is identifying data in the Supporting Information file “SOMAscan and RNAseq data for IL18 paper”. Due to the inclusion of these potentially identifying data, we have removed this file from your file inventory. Prior to sharing human research participant data, authors should consult with an ethics committee to ensure data are shared in accordance with participant consent and all applicable local laws.

Data sharing should never compromise participant privacy. It is therefore not appropriate to publicly share personally identifiable data on human research participants. The following are examples of data that should not be shared:

-Name, initials, physical address

-Ages more specific than whole numbers

-Internet protocol (IP) address

-Specific dates (birth dates, death dates, examination dates, etc.)

-Contact information such as phone number or email address

-Location data

-ID numbers that seem specific (long numbers, include initials, titled “Hospital ID”) rather than random (small numbers in numerical order)

Data that are not directly identifying may also be inappropriate to share, as in combination they can become identifying. For example, data collected from a small group of participants, vulnerable populations, or private groups should not be shared if they involve indirect identifiers (such as sex, ethnicity, location, etc.) that may risk the identification of study participants.

Additional guidance on preparing raw data for publication can be found in our Data Policy (https://journals.plos.org/plosone/s/data-availability#loc-human-research-participant-data-and-other-sensitive-data) and in the following article: http://www.bmj.com/content/340/bmj.c181.long.

Please remove or anonymize all personal information (<specific identifying information in file to be removed>), ensure that the data shared are in accordance with participant consent, and re-upload a fully anonymized data set. Please note that spreadsheet columns with personal information must be removed and not hidden as all hidden columns will appear in the published file.

3. Please remove all personal information, ensure that the data shared are in accordance with participant consent, and re-upload a fully anonymized data set.

Note: spreadsheet columns with personal information must be removed and not hidden as all hidden columns will appear in the published file.

Additional guidance on preparing raw data for publication can be found in our Data Policy (https://journals.plos.org/plosone/s/data-availability#loc-human-research-participant-data-and-other-sensitive-data) and in the following article: http://www.bmj.com/content/340/bmj.c181.long.

Thank you for this comment. However, there were NO identifying data in the Supporting information file. We assume that this was because there was a column called “Name” in one of the sheets, but the names were GDF001, GDF002 etc i.e. they were study identifiers not “personal names”. To ensure that this does not cause further confusion we have changed the column title to “Study number”.

**Additional Editor Comments:**

Dear Authors,
I appreciate your research on the immunological aspects in sarcopenia, looking at how proinflammatory interleukin-18 is linked to age-related muscle degeneration issues, and you have shown that the soluble IL-18 binding protein neutralization activity on IL-18, which in turn lowers inflammation-related consequences. The study further compared the IL-18bp and negative regulation of specific mitochondrial genes, which was revealed through the transcriptome analysis.

But here, perhaps you can talk about the negative enrichment of specific mitochondrial genes rather than directly saying it about the mitochondrial genes (as it is like globally in mitochondria).

Thank you for your insightful comments. We used the gene set approach as we think it gives a much better overall picture than looking at individual genes. We doubt that IL18 regulates individual mitochondrial genes and is more likely to have a generic effect on promoting an oxidative phenotype, this would appear most strongly in the gene sets rather than looking at individual genes. Furthermore, the most important aspect of this association is the fact that greater endurance is associated with greater mitochondrial content in muscle. However, to address your point, we have added a section looking at negatively associated mitochondrial genes with IL18BP. This section reads:

“*GSEA using the GO: Biological Processes gene sets also identified multiple components of mitochondria as negatively enriched with multiple aspects of mitochondrial biology showing a greater than 2-fold enrichment (Table S4). To confirm this analysis, we compared the genes that had a negative correlation coefficient with IL-18BP that was significant at a nominal p value of 0.05 (n=435) against the GO cell compartment division using DAVID functional annotation and also found over-representation of genes with a mitochondrial localisation (57 genes, p=3.6 x 10-6, Benjamini corrected p=0.0014). The most tightly correlated mitochondrially associated gene was TRMT10C a subunit of the mitochondrial RNAseP (r=-0.71, p=0.002). Other genes in this group included COX20, COQ3 and SDHAF3 and NDUFA4, all components of the electron transport chain or encode proteins required for its formation*.”

Most of the positive correlations between the parameters and IL-18bp are weak to very weak, and there are even negative correlations among the sarcopenia patients. Why didn't you take into consideration healthy individuals? Because it is always better to compare the normal individuals as well (also, you have mentioned we did not have a “control population”).

The study samples come from two separate clinical studies neither of which have a control group as neither study was set up to identify cross-sectional differences between control and disease but longitudinal differences in a population a) patients with aortic valve disease in response to surgery and b) individuals with sarcopenia in response to either Leucine or an ACE inhibitor or a placebo the LACE trial. In these studies, a control population is not needed as you are comparing before and after a treatment (a) or the effect of treatment against a placebo. There were therefore no samples from healthy individuals that could have been reasonably included.

Besides the most negative correlations, the SARC-F score was positively correlated with men and had a weak positive correlation in women, and why the SARC-F follow-up was not assessed after 6 months and 12 months is not shown in Table 2.

Thank you, SARC-F was not followed up because it was a recruitment/screening criterion for the LACE trial and not an outcome measure.

We have added the following comment to cover this point:

“SARC-F was recorded as part of the initial screening for sarcopenia with a minimum score of 3 used to take individuals through to randomisation.”

It would have been better if you could have added the regression lines of the correlations from the correlation figures. Thus, the IL-18BP with the SARC-F score for men could be a potential marker for sarcopenia due to positive correlation.

We have added regression lines where the correlations are Pearson or bi-weight mid-correlations. However, where the figures are Spearman’s correlations (rank based) the correlation coefficients reflect closeness to a defined order where the correlation is monophasic but does not need to be linear. As a result, we feel that regression lines can be mis-interpreted as indicating a linear association even where that is not the case.

I have some additional suggestions and comments

You have mentioned that the surgery increased and decreased the concentrations of 295 and 301 proteins, respectively; the IL-18bp is the one among them, and it is a potential factor against the loss of strength from the muscles of the sarcopenic patients.

Could you please slightly explain about the other proteins if there is information? Besides the cells of the immune system and muscle cells, do you expect any other non-haematopoietic cells to express these proteins?

Is there a contribution of cells from other tissues concerning these proteins? Does the SOMAscan identify the proteins from specific tissues?

The SOMAscan assay we used measures 1300 different proteins in a biological sample (in our case plasma). The proteins are a pre-defined set (it is equivalent to running 1300 ELISAs on a single sample). The system does not differentiate the proteins based on their tissue source so other than for proteins that are only expressed in one tissue it is not possible to know what cell-type(s) made the protein. The assay does contain some tissue restricted proteins where it is possible to hazard a guess at the tissue source but for most proteins in our blood samples, which cell types and the relative contribution of each cell type to the amount of each protein is not knowable.

As originally indicated in the text, the analysis of the protein changes is large, and we have written a separate paper covering this analysis. The major focus of that paper is the loss of muscle mass rather than strength as these are separable observations in the aortic surgery model. As we identified the IL-18/IL-18-BP/IL-37 axis that associated well with the loss of strength in one situation we felt that these were of sufficient interest and complexity to warrant a separate manuscript.

We have therefore added the following to the results

*“Of the proteins that increased in expression after surgery, 49 had a positive association with loss of hand grip strength whereas 67 had a negative association. Both positively and negatively associated proteins came from a range of different protein groups and included both intracellular and extracellular proteins. The associations of intracellular proteins with change in muscle strength are difficult to rationalise but are likely to reflect tissue breakdown. Of more interest therefore are known signalling proteins and their regulators. The SOMAscan assay showed a reduction in myostatin following surgery, as well as an increase in GDF-15 both of which were consistent with our previous ELISA based analysis[28]. Of these, post-surgery myostatin had a negative association with loss of strength (patients with higher myostatin levels on post-surgery lost least strength by day 7, Table S3) whereas day 1 GDF-15 had a positive association with loss of strength (patients with higher GDF-15 levels on day 1 lost most strength by day 7, Table S3). Other changes of potential interest include the increase in 3 IGF binding proteins (IGFBP1, IGFBP4 and IGFBP5) all of which also positively associate with loss of strength consistent with a role for IGF1 in the maintenance of strength even though IGF1 itself did not change significantly following surgery in this study.”*

Could you please provide the source link for the SOMAscan?

We are not sure what you mean by this. SOMAscan is a proprietary method to measure proteins produced by the company Somalogic. In this assay they use aptamers to bind to proteins and quantify the binding using a microarray approach.

We have added (Boulder, Colorado, USA) as that is the place that the assays were performed.

Lack of balance between the IL-18 and IL-18bp is the main factor behind sarcopenia with a loss of muscle mass and strength. Besides, did you compare this factor with any other diseases that are directly or indirectly related to sarcopenia?

We have only analysed patients with aortic valve disease undergoing aortic surgery and individuals with sarcopenia. We would need to obtain samples from other conditions along with the appropriate ethical approval to look at those specifically and this is beyond our current capability.

The elevation of IL-18 with individuals with sedentary lifestyles and old populations is generally observed. Also, a similar finding was done before with IL-12, also for the association with sarcopenia (https://pmc.ncbi.nlm.nih.gov/articles/PMC8140914/), so how do you compare other interleukins? Do you consider IL-18 the major contributor cytokine to sarcopenia? Perhaps the expression of the proinflammatory factors commonly triggered by a sedentary lifestyle, etc., and maybe more gene networks and other transcription factors are behind it. So how do you infer this?

Though one would expect that the LACE sarcopenic individuals were less active we don’t have activity data to know whether that is true, so it is not possible to introduce physical activity levels other than as a theoretical discussion point.

We have measurements in the LACE Sarcopenia cohort for IL-6 but not for other interleukins. In these individuals IL-6 does not show any associations with strength or other muscle physiological parameters.

In the Aortic surgery cohort, we have the measurements of other interleukins in the SOMAscan data set but not measured any other way and so not in the whole cohort. In the SOMAscan set, IL18BP was the protein most tightly associated with maintenance of strength (r=-0.73, p=3x10­^-6^, q=0.001). There are 61 other interleukins, interleukin receptors or associated proteins that are measured in the SOMAscan assay. Of those the only ones that show an association with the maintenance of strength at a q value (FDR) significance p<0.05 were IL10RA (r=-0.564, p=0.001, q=0.017), IL18RAP (r=-0.568, p=0.001, q=0.017), IL37 (r=-0.532, p=0.002, q=0.022), IL6ST r=-0.5, p=0.004, q=0.028), IL7 (r=-0.481, p=0.007, q=0.034), IL4R (r=-0.471, p=0.009, q=0.039), IL2RG, r=0.463, p=0.01, q=0.04), IL2(r=-0.455, p=0.011, q=0.04, IL20 (r=0.452, p=0.012, q=0.046). Thus, three of the 4 proteins most closely associated with loss of strength following surgery were from the IL18/IL37 system and all showed a negative association with loss of strength. We have added a comment to this effect to the results. All of these, except IL20 and IL2RG, are negative associations. A negative association would suggest a functional role for the IL18 antagonists and IL10 receptor but for the others a positive association would be more indicative of the function in muscle wasting as they are pro-inflammatory factors

We have added a paragraph discussing this information to the results and a comment in the discussion.

The results paragraph reads

“*IL-18BP was not the only interleukin, interleukin receptor or interleukin binding protein in the day 1 SOMAscan set to associate with change in grip-strength at an FDR q<0.05. The others were IL-10RA (r=-0.564, p=0.001, q=0.017), IL-18RAP (r=-0.568, p=0.001, q=0.017), IL-37 (r=-0.532, p=0.002, q=0.022 though we were subsequently unable to verify this analysis by ELISA), IL-6ST r=-0.5, p=0.004, q=0.028), IL-7 (r=-0.481, p=0.007, q=0.034), IL-4R (r=-0.471, p=0.009, q=0.039), IL-2RG, r=0.463, p=0.01, q=0.04), IL-2(r=-0.455, p=0.011, q=0.04, IL-20 (r=0.452, p=0.012, q=0.046).”*

Please note we have indicated that the IL-37 data was not reproducible comparing IL-37 quantified by SOMAscan to ELISA. We therefore note elsewhere in the document the following:

“*Of note, IL-18 was not reported in the SOMAscan analysis. IL-37 measured by ELISA did not match IL-37 measured by SOMAscan, however, given the use of the IL-37 ELISA in other publications, the ability to measure IL-37 in a larger number of samples and the fact that our median IL-37 measured by the ELISA (66.6 pg/mL) was in the range reported elsewhere (see [31] for meta-analysis) we only report comparison of the IL-37 ELISA data with strength.”*

Please mention the expansion for the abbreviations at least one time in the introduction or elsewhere; for instance, please mention the expansion of LACE concerning your study (Leucine or Angiotensin Converting Enzyme inhibitors for sarcopenia). There are other expansions also: the Laparoscopic Approach to Cancer of the Endometrium (LACE) trial. So a reader who is not an expert in sarcopenia may not immediately recognise the term unless you mention it. So please mention the expansion.

We apologise for this oversight. We had thought that the sentence in which it appeared made it clear but we have added that to the abstract and the Methods section.

There must be proinflammatory inhibitor drugs available for those who have undergone aortic surgery, and these drugs may act like IL-18bp to neutralize IL-18, so do you have an idea to compare them with your study now or with your future studies?
It would be interesting to use an anti-IL-18 neutralising antibody or indeed a recombinant IL-18BP prior to surgery (we are unaware of any selective small molecule anti-inflammatories that just target IL-18), this is not something we are currently thinking about but would need a new grant proposal and ethical approval.

Achison et al., 2020 (https://pmc.ncbi.nlm.nih.gov/articles/PMC8977979/) found there is no efficacy of ACE inhibitors or leucine for the muscle mass or any improvement associated with sarcopenia, so why did you choose? Is there any report shows the association prior to Achison et al., 2020 et al., study?

You are correct that neither ACEi nor leucine improved muscle strength in the LACE trial. However, this is not relevant to the current analysis; indeed, the lack of effect makes it possible to analyse alternative mechanisms such as the IL-18/IL-37 axis without interference from a potentially efficacious therapy. The key reason for selecting this population to study is that they comprise one of the few available consistently phenotyped populations with sarcopenia, with repeated measures, and with repeated biobanked samples for analysis. Some of the analysis we have carried out subsequent to the trial is trying to determine why the study did not show any benefit of either the ACE inhibitor or Leucine by analysing subgroups, but we are limited by the size of the study (recruitment proved much more difficult than anticipated).

The choice of both leucine and ACE inhibitor for the LACE was based on prior studies that raised the possibility that either leucine or and ACE inhibitor may be useful. For example, there are data showing older people taking ACE inhibitors have better preservation of walking speed, and small randomised control trials had also suggested that ACE inhibitors may be useful (e.g. PMID: 17923654). Meta-analysis of such trials is included in the Achison paper which details some of the other studies. It is difficult to know how to include this information in the current paper, but it is covered extensively in the meta-analysis we published in Achison et al.

Gene enrichment negative associations with circulating IL-18BP show the negative association with genes involved in different metabolic pathways, including oxidative phosphorylation, IFN-gamma response, etc.; however, it didn't show any specific genes with negative enrichment besides the pathway. It would be better to describe the specific genes from the gene enrichment analysis.

As discussed above (first response), we think that understanding the effect on a pathway is more informative of an overall function. See for example the rationale given in the GSEA paper we reference. We have however added some comment on specific genes as described above.

How did you choose 8.48 ng/mL of plasma IL-18bp as the median point for comparing the ARC-F score, QMVC, etc.? Whether any previous study standardized the specific point (8.48 ng/mL) of the measurements or the median point is based on your study/trial only; for instance, you have mentioned "SARC-F score (A) was higher in those with IL-18BP > 8.48 ng/mL" in accordance with Figure 3.

8.48ng/mL was calculated as the median plasma concentration for the plasma samples of our study. It gave us the ability to split the cohort into higher than median and lower than median IL18BP to see if there was any effect of different IL18BP levels. There is as far as we are aware no other similar analysis in the literature. The rationale for using the median is that plasma IL18BP levels are not normally distributed. The mean value is overly affected by a few individuals with high IL18BP levels so using the mean would bias the numbers of individuals in each group with significantly more in the lower IL18BP group.

Seems the Figure 3 with IL-18BP < 8.48 having some skewed distribution, have you normalized the data using log or square root transformation prior to the analysis concerning the plasma IL-18BP data?
As indicated above this is the reason for using the median value. It is also why we used non-parametric statistics for the analysis as these are rank based rather than based on the absolute values. Transformation does not change Spearman’s correlation coefficients or significance, nor does it change significances in difference calculated by Man-Whitney U test as the rank order of the variables is retained and that is what these tests use. Transformation does allow the use of parametric stats (t-test and Pearson correlation) if the resulting distributions are normal. However, in our study neither transformation produces a normal distribution for IL18BP so there is no benefit in carrying out the transformations as the underlying requirements of the parametric tests are failed.

Your study shows IL-18 has no association with muscle strength as expected, as it acts as a proinflammatory factor; however, 6MWD (walking) shows a positive correlation with IL-18 in men, not in women. It means physical activity (walking) is inducing the proinflammatory cytokine (IL-8) in man? Please clarify.

Yours is an interesting inference, but we do not think we can assume that the association of IL-18 and 6MWD means that IL-18 is increased by walking. There are a number of reasons for this. Firstly, the 6MWD test does not test physical activity on a day-to-day basis (though there is likely to be some association) it tests the distance someone can walk in 6 minutes at a specific time. Secondly, the blood samples were taken before the walking test was undertaken so the activity of the walking test could not have contributed to the plasma levels. Finally, and probably most importantly, there is no association of total IL-18 with any physiological parameter it is only an association of 6MW with free IL-18. As free IL-18 is a calculated value from total IL-18 (which does not correlate) and IL-18BP which does correlate the association is driven by the amount of IL-18BP not IL-18. In fact the association of free IL-18 is only marginally stronger (probably not significantly) than the association with IL-18BP, therefore it seems reasonable to conclude that IL-18BP is the factor driving the association.

Our view, is that if IL-18BP regulation of IL-18 is key to its function in muscle, IL-18 contributes to muscle homeostasis in two ways: under basal conditions (low levels of free IL-18) or during development it promotes an oxidative phenotype (perhaps via fibre-type) and increases endurance, whether this is through direct effects on muscle cells or through influencing satellite cell differentiation is unknown. As the 6MW test is in part a test of endurance the negative association of 6MW distance with IL-18BP is a reflection of this long-term effect on mitochondrial function and oxidative capacity. This suggestion is consistent with our data and with data from IL-18R knockout mice and patients with HIV described in the paper. An alterative situation is that IL-18BP has a separate unknown effect on muscle cells or that it is just a bystander/reporter

We hope that changes the discussion section entitled “Under normal physiological conditions” clarify this and include the following changes:

*“Maintenance of mitochondrial function or a greater mitochondrial number is associated with greater physical performance and with the preservation of muscle mass, with mitochondrial dysfunction identified as a component of muscle loss and poor physical performance in old age [39], chronic disease [40, 41] and in patients undergoing surgery [42]. These factors fit with the inverse association of IL-18BP with mitochondrial gene expression and with better physical performance in our cohorts.”*

and

“A third possibility is that the associations of IL-18BP with strength are independent of IL-18/IL-37 either because IL18-BP is acting in an, as yet, unknown way or because it is reflective of a greater response in some individuals to switch off the inflammatory system by one or more mechanisms rather than having a direct effect”.

However, in the inflammatory condition its pro-inflammatory function predominates. The fact that IL-18 did not change in response to surgery suggests that the milieu in which it is working is important (i.e. it is acting as a co-inflammatory agent rather than as a single factor). We have added a comment to discuss this point.

This comment reads

*“However, the lack of change in circulating IL-18 levels in response to surgery argues against an independent role for IL-18 in the loss of strength after surgery. Therefore, if IL-18 is contributing to the loss of strength, it will be by acting synergistically with inflammatory cytokines that do increase in response to surgery (e.g. IL-6, IL-24). Alternatively, IL-18BP could be acting as a surrogate measure of the natural inactivation of the inflammatory response to surgery, where those who do this fastest or to the greatest extent lose least strength. It is known that inactivating inflammatory responses is critical, and that IL-18BP is involved to some extent as reduced inactivation of IL-18 by IL-18BP is a key component of macrophage activation syndrome[20].”*

IL-18 is also associated with several autoimmune diseases, and there are reports showing the isoforms of IL-18bp (https://pmc.ncbi.nlm.nih.gov/articles/PMC15564/), so referring to these kinds of studies may provide a better clue. IL-18 is known to induce IFN-γ production there by inducing the Th1 cells. So, it is better to describe how the IL-18 specifically acts on muscle cells with sarcopenia, thereby reducing the mass of the tissues and strength.

It is difficult to know what role the induction of IFN-γ by IL-18 plays in either model and as a consequence in the regulation of strength in these conditions. Neither IL-18 nor IFN-γ change in response to surgery. Furthermore, as IL-18BP increases following surgery in all patients free IL1-8 goes down. Consequently, IL-18 signalling should go down. This does mean that in the acute loss of strength following surgery any effects of IL1-8 are likely to be co-stimulatory in some way (i.e something (e.g. IL-6 is increased by surgery and in those with higher free IL-18 it has a bigger effect) to investigate this a little further we investigated the SOMAscan data. The interleukin would have to increase to have the proposed effect, we therefore identified those that increased significantly (IL-1b, IL-2, IL-6, IL-11, IL-16, and IL-24) then reduced this selection to those that also associated with loss of hand grip strength by Pearson correlation (IL-6 r^2^ = 0.16, and IL-24 r^2^= 0.41, note this figure is inflated by an outlier so is not as strong in the bi-weight mid correlation analysis). Combining each with IL-18BP (r^2^= 0.40) independently in a multiple linear regression against hand grip strength showed that both increases r^2^ for the correlation (to IL-6 + IL-18BP r^2^=0.51 and IL-24 + IL-18BP r^2^ =0.55). Furthermore, we used the equation from the multiple regression with handgrip data to calculate composite variables and these composite variables also had better correlations with loss of quadriceps strength [IL-18BP r^2^= 0.16 (p=0.04), IL-6 r^2^=0.09, (p=0.115), IL-24 r^2^= 0.14 (p=0.055), IL-18BP + IL-6 r^2^=0.28 (p=0.005), IL-18BP + IL-24 r^2^= 0.23 (p=0.011)]. These data raise the possibility that IL-18 synergises with either IL-6 or IL-24 to promote loss of strength in response to surgery.

We have added a comment to the discussion to raise the possibility of synergism between the ILs but have not gone into the detail given its speculative nature. Given the difference in the associations of IL-18BP with strength loss in the acute state and with strength in the chronic states, we have not explored this by ELISA as it is unlikely to yield useful information, and we do not have capacity to do it)

The added section reads

“However, the lack of change in circulating IL-18 levels in response to surgery argues against an independent role for IL-18 in the loss of strength after surgery. Therefore, if IL-18 is contributing to the loss of strength, it will need to be acting synergistically with inflammatory cytokines that do increase in response to surgery (e.g. IL-6, IL-24).”

Your article is written well; however, it needs to be more precise. Please submit the RNA data set to NCBI. Have you compared the Gene Expression Omnibus? It may provide additional evidence of the gene expression, especially when dealing with mitochondrial genes. So please address the comments of the reviewers and mine, and revise the manuscript accordingly.

 The RNA data used in this study are a subset of a data set that has been submitted to NCBI as part of a study currently under review looking at factors that affect gene expression in response to surgery. As that larger study is not yet reviewed and using the GSE number in this paper would release the data before it was normally published, we hoped that including the processed data in this paper as a supplement would allow us to maintain the privacy of the main dataset for the time being. We hope that you can understand this as we have included all the data used to generate this paper here so as not to delay this publication.

We are not sure what you mean by comparing the data with the Gene expression omnibus (GEO) and what it would show. Our understanding is that GEO is simply the repository where NCBI curate the submitted data, and we are unclear how we would compare our data with that or what it would tell us.

If you mean comparison with the Gene ontology terms in for example DAVID (or GSEA which we used) then it depends on which division of GO terms you mean. When we use GO:BP the biological processes division in GSEA then it effectively produces the same result that we get using the Hallmark gene sets except there are many more overlapping gene sets. So, for example, you get the following table which we have added to the paper.

| NAME | SIZE | ES | NES | NOM p-val | FDR q-val |
| --- | --- | --- | --- | --- | --- |
| GOBP ATP SYNTHESIS COUPLED ELECTRON TRANSPORT | 82 | -0.599 | -2.76 | 0.000 | 0.000 |
| GOBP MITOCHONDRIAL ELECTRON TRANSPORT NADH TO UBIQUINONE | 43 | -0.637 | -2.52 | 0.000 | 0.000 |
| GOBP AEROBIC RESPIRATION | 147 | -0.492 | -2.50 | 0.000 | 0.000 |
| GOBP OXIDATIVE PHOSPHORYLATION | 109 | -0.512 | -2.45 | 0.000 | 0.000 |
| GOBP ELECTRON TRANSPORT CHAIN | 104 | -0.509 | -2.43 | 0.000 | 0.000 |
| GOBP PROTON MOTIVE FORCE DRIVEN ATP SYNTHESIS | 45 | -0.608 | -2.36 | 0.000 | 6*10^-04^ |
| GOBP CELLULAR RESPIRATION | 182 | -0.436 | -2.27 | 0.000 | 0.001 |
| GOBP ATP BIOSYNTHETIC PROCESS | 65 | -0.505 | -2.19 | 0.000 | 0.005 |
| GOBP MITOCHONDRIAL RESPIRATORY CHAIN COMPLEX ASSEMBLY | 85 | -0.478 | -2.19 | 0.000 | 0.005 |
| GOBP MITOCHONDRIAL ELECTRON TRANSPORT CYTOCHROME C TO OXYGEN | 16 | -0.694 | -2.15 | 0.002 | 0.008 |
| GOBP TRICARBOXYLIC ACID CYCLE | 27 | -0.603 | -2.13 | 0.000 | 0.010 |
| GOBP NADH DEHYDROGENASE COMPLEX ASSEMBLY | 35 | -0.559 | -2.09 | 0.002 | 0.015 |
| GOBP TRANSFORMING GROWTH FACTOR BETA PRODUCTION | 29 | -0.563 | -2.07 | 0.000 | 0.017 |
| GOBP HOMOLOGOUS RECOMBINATION | 39 | -0.514 | -2.01 | 0.000 | 0.031 |

Key

Size= number of genes in the gene set

ES =enrichment score

NES = Net enrichment score (enrichment score normalised for gene set size)

As you will see, the top associations are all different but overlapping aspects of mitochondria and are likely to reflect an approximate 2-3-fold enrichment in mitochondrial genes that negatively correlate with IL-18BP pre-surgery. If you look at the genes involved in driving the enrichments with these sets, they cover several different parts of mitochondrial biology with components of all electron transport complexes present suggesting again that it is not an effect on one or two genes but a pathway effect.

As described above, we have also added an additional section covering analysis of genes with a significant negative correlation with loss of strength through the DAVID gene ontology and again shown enrichment for genes associated with mitochondria.

[Note: HTML markup is below. Please do not edit.]

Reviewers' comments:

Reviewer's Responses to Questions

**Comments to the Author**

1. Is the manuscript technically sound, and do the data support the conclusions?

The manuscript must describe a technically sound piece of scientific research with data that supports the conclusions. Experiments must have been conducted rigorously, with appropriate controls, replication, and sample sizes. The conclusions must be drawn appropriately based on the data presented.

Reviewer #1: Yes

Reviewer #2: Partly

2. Has the statistical analysis been performed appropriately and rigorously?

Reviewer #1: Yes

Reviewer #2: Yes

3. Have the authors made all data underlying the findings in their manuscript fully available?

The [PLOS Data policy](http://track.editorialmanager.com/CL0/http:%2F%2Fwww.plosone.org%2Fstatic%2Fpolicies.action%23sharing/1/010f019790f03fb6-aaab71c6-ed23-4f38-b631-66daf8f856be-000000/4821e45XbjubaQZi2-GgseRrkr_3x55dT2HU3IjqM40=216) requires authors to make all data underlying the findings described in their manuscript fully available without restriction, with rare exception (please refer to the Data Availability Statement in the manuscript PDF file). The data should be provided as part of the manuscript or its supporting information, or deposited to a public repository. For example, in addition to summary statistics, the data points behind means, medians and variance measures should be available. If there are restrictions on publicly sharing data—e.g. participant privacy or use of data from a third party—those must be specified.

Reviewer #1: Yes

Reviewer #2: Yes

4. Is the manuscript presented in an intelligible fashion and written in standard English?

PLOS ONE does not copyedit accepted manuscripts, so the language in submitted articles must be clear, correct, and unambiguous. Any typographical or grammatical errors should be corrected at revision, so please note any specific errors here.

Reviewer #1: Yes

Reviewer #2: Yes

5. Review Comments to the Author

Please use the space provided to explain your answers to the questions above. You may also include additional comments for the author, including concerns about dual publication, research ethics, or publication ethics. (Please upload your review as an attachment if it exceeds 20,000 characters)

**Reviewer #1:** Thank you for submitting your manuscript entitled "IL-18 BP, a biomarker of strength maintenance after surgery but reduced physical performance in age - related sarcopenia" to our journal. The combination of proteomic screening and subsequent validation in two different cohorts provides a comprehensive approach to understanding the role of IL-18BP.
Weaknesses and Suggested Revisions:
1. The introduction could benefit from a more detailed discussion of the existing literature on IL-18BP and its ligands in the context of muscle wasting. This would help to better justify the hypothesis and provide a clearer rationale for the study.

We have expanded the section describing IL-18 in the Introduction, but we are not sure that it helps with the rationale for the study. The initial rationale was to examine a broad range of proteins that associate with loss of muscle strength using an ‘omics approach rather than to examine IL-18 so we would be generating a false hypothesis that required some of the results to justify it.

Effectively our hypothesis was ‘Do any of the 1300 proteins in the SOMAscan data set associate with the loss of muscle strength’. The answer was yes STC1 (or whatever STC1 measured by SOMAscan is) and IL-18BP among others, and the rest of the paper is investigating that finding in a second setting by looking at individuals who were sarcopenic and then looking back to see if differences we had seen were consistent.

2. The description of the SOMAscan assay and ELISA analysis lacks sufficient comprehensiveness and is overly concise.

It is not possible to give more detail on the SOMAscan assays as they were performed by Somalogic using their proprietary system that was available at the time. Essentially, we sent them plasma samples, and they returned the data to us as a spread sheet. This was only available as a commercial service using a proprietary technology.

We have added a short description of the ELISA protocol

3. In the RNAseq analysis section, it would be beneficial to specify the number of genes analyzed and the criteria used for filtering genes before correlation analysis.

We have added a section covering the number of genes and the selection process. As a result of the correlation approach and the fact that we were using WGCNA to carry out the analysis, any gene had to be detected in all samples. This restricted the total number to 15071 sequences. We have added this information to the methods which now includes the comment

“*The data set was trimmed to genes that were detected in all samples leaving 15071 transcripts.*”

4. The discussion touches on the potential mechanisms underlying the associations observed, but it could be expanded to more thoroughly explore the biological plausibility of the findings.

We have modified the discussion to cover this as well as we can, hopefully, without over-speculating. Please see Discussion: “in response to surgery” end of paragraph 1 and “under normal physiological conditions” paragraphs 2 and 3.

**Reviewer #2:** Summary:
This study primarily aimed at identifying biomarkers of acute and chronic sarcopenia, a condition characterized by loss of muscle mass and/or function. The authors began by examining changes in plasma proteins in relation to muscle mass or function loss in a cohort of patients who underwent aortic surgery, which is a well-accepted model for acute sarcopenia. They report approximately 600 proteins being up- or downregulated 24 hours post-surgery. Through correlation analyses, they uncovered IL-18 binding protein (IL-18BP) as being positively associated with muscle function preservation in the grip strength test performed 7 days post-surgery. To ascertain the validity of those results, they performed ELISA assays to quantify IL-18BP as well as IL-18. To determine whether IL-18BP may also serve as a biomarker for chronic sarcopenia, they revisited a previously generated dataset from a separate cohort of patients affected with chronic sarcopenia to determine whether there was a similar correlation between IL-18BP and functional data. They found that, unlike in the aortic surgery patients (acute sarcopenia), IL-18BP levels were negatively associated with muscle function.
Strengths:
Sarcopenia is a generalized disorder characterized by a significant loss in skeletal muscle mass and/or function, which leads to increased risk of noncommunicable diseases and mortality. With the sedentary lifestyle crisis, sarcopenia is a significant contributor to the global burden of disease, and understanding the mechanisms driving this process or identifying biomarkers that could predict the onset or the progression is extremely relevant to human health.
Biomarkers that do not require invasive techniques are ideal for a translational approach, especially when linked to functional outcomes that can be employed in the clinic.
The rationale for the study was well presented. The hypothesis was clearly stated. The techniques and statistical approaches were appropriate and rigorous.

Potential areas for development:
While these results are extremely relevant to the field, the manuscript is currently lacking a concise overview of the mechanisms contributing to “acute” versus “chronic” sarcopenia. Recovery from invasive surgery and chronic sarcopenia, such as during aging, are distinct physiological states differentially impacting muscle structure and function. While inflammation is an unequivocal contributor to both conditions, it is also important to consider that the effects of pro-inflammatory cytokines are heavily dependent on the timing, location, and duration of stimuli.

We have not included an overview of mechanisms contributing to acute and chronic sarcopenia for a couple of reasons. Firstly, the paper is already quite long and an additional section covering such a large topic is difficult to include. Secondly, we are only looking at strength and not muscle loss making it more difficult to know exactly what to include again without it becoming overlong. We have however, expanded our discussion of the different mechanisms by which IL-18BP may contribute to strength maintenance following surgery but limit the maintenance of mitochondrial function in the chronic situation. We hope that this is acceptable.

From an Exercise Physiologist’s standpoint, inflammation is a crucial response facilitating muscle injury repair. In fact, transient elevation of pro-inflammatory cytokines is a normal response to exercise. A good example is IL-6, a pro-inflammatory cytokine that has been categorized as a top “Exerkine” and a “Myokine”. Upon acute injury or resistance exercise, skeletal muscle produces IL-6 to activate satellite cells (muscle stem cells). This is a fundamental mechanism for muscle recovery, and this is why inflammation is essential after exercise training or acute muscle injury. What may cause a lot of confusion in the scientific community is the fact that, while the transient release of pro-inflammatory factors is beneficial, chronic elevation of these factors becomes pathologic and can cause the opposite effects. (See: https://www.sciencedirect.com/science/article/pii/S155041310700366X and https://pmc.ncbi.nlm.nih.gov/articles/PMC8306542/).

Thank you, we have expanded on the role of interleukins in sarcopenia and in hypertrophy to hopefully cover this. These additions are in paragraphs 3 and 4 of the revised Introduction.

Given the contrasting results presented in the paper between the acute versus chronic models of sarcopenia, it is important to discuss these concepts. This may also help with the interpretation of the results. Including one paragraph on the role of inflammation in skeletal muscle physiology would be very helpful.

While we have not added a discussion of acute vs chronic sarcopenia for the reasons given above, we have added a section examining the role of inflammation in muscle physiology (paragraphs 3 and 4 of the introduction).

The authors found very interesting results on IL-18/IL-18BP in these 2 cohorts of patients. IL-18 signaling in skeletal muscle has documented effects that are not reported or discussed in the paper such as induction of AMPk. A more thorough review of literature on this topic is highly recommended. Additional information on IL-18 may be included after the additional paragraph on inflammation and skeletal muscle.
Here are some references to look at:
https://pmc.ncbi.nlm.nih.gov/articles/PMC3749341/
https://www.frontiersin.org/journals/endocrinology/articles/10.3389/fendo.2022.971745/full
There is also a need to improve clarity and conciseness. I provided several examples below, but there are more, and I would encourage a senior co-author or collaborator to proofread before resubmission.
General minor comments:
1) The term gender should be avoided and updated to sex. (Using the find function, I found 4 occurrences: p. 9, 11 (2X), and 21.

We used ‘gender’ because it was given as a response to a question by the individual and was not determined by karyotyping. Whilst in the vast majority of cases (probably all) we expect the answer given to be the individual’s biological sex, but we cannot be sure that this is the case. As a result, gender more accurately reflects the variable we are looking at. We have therefore replaced the 2 instances where we referred incorrectly to ’sex’ with ‘gender’.

2) Please, use the same terminology throughout the manuscript (Aortic surgery or Acute Sarcopenia). The term “acute sarcopenia” is appropriate and may contribute to better manuscript cohesion.

We have unified the description of the cohort of individuals who had surgery to read as the Acute sarcopenia cohort. We have retained the use of pre- and post-surgery and similar wording where it is relevant to describe the methodology used or the time frame of the analysis.

3) There are several double negative statements, which can become difficult to understand. An example is provided below. The authors should review the entire manuscript with this vision.

We have been through the manuscript to simplify the language where it is possible.

4) Data generated from a previously published clinical trial were analyzed from a different perspective. Therefore, terms like “re-examined” or “re-analyzed” could be updated to “examined” and “analyzed”… a previously published dataset.

We have made these changes

Specific Comments:
Abstract:
P.2. – Line 11: Please clarify what the LACE trial is at the first occurrence.

This has been clarified in the abstract and in the main text.

P.2. – Line 4-5: Clarity: “We used a proteomic screen of plasma to identify proteins associated with strength loss.” Since you are also reporting a positive association with skeletal muscle function, the statement should be updated to “we used a proteomic screen of plasma to identify proteins associated with changes in strength”.
Given that in the short period following surgery patients only lose strength we thought that the original was accurate but have changed it accordingly.

P.2 – Line 8-9: Avoid speculation: “Analysis of the day 1 protein levels identified IL-18BP as a potential protective factor against loss of strength.”
The term “protective” may be speculative. I would recommend updating to “associated”. Also, two negatives in a sentence can become confusing (against, loss). Perhaps, rephrasing to something like: “Analysis of the day 1 protein levels identified IL-18BP as being positively correlated with the maintenance of grip strength”.

We have made this change.

P.2. – Line 19: Contrasting wording: “negative enrichment for mitochondrial genes”. It is unclear what this means. Are you referring to DEGs (Differentially expressed genes)? If so, please use universal bioinformatic terminology (Up/Down; or Upregulated/Downregulated).

Negative enrichment is a specific term related to how gene set enrichment analysis works. The approach takes a ranked set of genes (in our case ranked by correlation coefficient) and compares the order of genes from defined gene sets (e.g., a gene set associated with oxidative phosphorylation or inflammatory processes etc) that is observed in the data with a number of randomised lists of the same genes (we use the standard 1000 iterations) and determines the likelihood of that order appearing by chance. The output is a net enrichment score (enrichment normalised for gene set size) together with a set of statistical outputs. We choose the FDR and set a threshold of 0.05 for things that may be important. What positive enrichment means in this context would be there are more genes than would be expected from the gene set listed in the leading edge (genes towards the top of the ranking) of the ranked list. Negative enrichment therefore is there are more genes than expected at the trailing edge of the ranked list.

We have added a comment on gene set enrichment to the methods to explain this.

“*GSEA compares the order of genes from pre-defined gene groups (gene sets) in the ranked list with the order found in randomised lists (we used the standard 1000 iterations) and computes the relative enrichment at either end of the list for that group and the likelihood of that enrichment occurring by chance.”*

Introduction:
I gave general recommendations in the “Potential areas for development” section above. I will provide specific feedback in the revised version of the manuscript.
Methods:
P5. Line 11-12: Please rephrase this statement and specify the muscle loss threshold at which patients were classified as sarcopenic. Alternatively, you may want to simply update “muscle loss” to “muscle cross-sectional area”.

Thank you for pointing this out. In previous studies using this and other cohorts we have used 10% RFcsa loss by day 7 to identify muscle wasting in the surgery patients but, as we don’t separate the patients in this study, we have removed the reference.

Corrected

Results:
P.8- Line 17-18: Citation: “A more complete analysis of these changes will be described
Elsewhere.” Did you mean that these were previously published? Unless a reference can be provided, this statement should be removed since it’s in the future.
We have written a second paper looking specifically at the patients undergoing aortic surgery. This paper details the changes in proteins associated with muscle loss (rather than loss of strength) in more detail. Combining these data into one paper would produce an overly complex paper without a clear message. Hence, we thought it better to divide the analysis into two separate papers (this one is strength). As the other paper has more analysis and a separate set of proteins, it has taken longer to complete the studies and write. We thought that including this statement would allow people to know that there was a more complete analysis, and so allow this paper to be linked to the other when it is published. However, we have removed the statement.

P.9 Line 5- “Human surgical model” should be updated to “Human patient population” or “cohort of patients”.

This line now reads

*“*The above data suggested that higher levels of IL-18BP following surgery associate with the maintenance of strength following surgery.”

P.9 – Line 10-15: Clarity: I had to re-read to notice the difference between the first and second sentences. Beginning sentences by stating the time point would help with clarity.

Thank you, for pointing this out, we have changed this now to read:

In men, at baseline, IL-18BP was inversely associated with both grip strength (r=-0.314, p=0.014 Fig 2A), quadriceps maximum voluntary contraction (QMVC, r=-0.335, p=0.014, Fig 2B) and six-minute walk distance (6MWD, r=-0.359, p=0.005, Fig 2C). Similar associations of IL-18BP with performance were seen in men at 6 months [ IL-18BP vs grip strength (r=-0.442, p=0.003), QMVC (r=-0.330, p=0.025) and 6MWD (r=-0.447, p=0.003) (Figs 2D-F)] and at 12 months [IL-18BP vs grip strength (r=-0.403, p=0.007) and 6MWD (r=-0.404, p=0.005) (Table 2). In women, there were no associations between IL-18BP and physical performance at base line but there was a negative association with QMVC at 6 (r=-0.442, p=0.003) and 12 months (r=-0.355, p=0.038, Table 2).

P.9- Line 8-22: Clarity: Given that males and females were analyzed separately, including results from both sexes in a single sentence can become confusing. I would recommend presenting the results in separate sentences and avoiding comparing the results. For example: update “In men but not women,” to “In men,”.

Please see the modification above

P. 10 – Line 7-10: This sentence is very difficult to understand and unclear. Negatively associated with what? Perhaps breaking down this sentence into 2 sentences would help with clarity.

Thank you for this. It is true that we had included both men and women in this analysis when we had specifically not done that for the LACE cohort. We have now removed the analysis including both groups and the section now reads

“*This analysis showed that consistent with the analysis of the sarcopenia cohort, in men IL-18BP negatively associated with QMVC (Fig. 4), there were too few women in this study to allow sensible analysis.”*

We have also modified the figure accordingly

P. 10 – Line 17: Contrasting wording: “negatively enriched”

Please see explanation above

P.10 – Line 22-24: Terminology: Since the data reported is limited to basic muscle function tests and imaging, the term physiology is too broad. The words “morphology and function” would be more appropriate.

We have replaced the term ‘physiology’ with ‘strength’ in 3 places where that is what is analysed and with ‘muscle mass and strength’ where the sentence is broader.

Discussion:
As this section may require intensive recrafting to address my comments, I will provide additional feedback when receiving the revised manuscript.

Best of luck!

6. PLOS authors have the option to publish the peer review history of their article ([what does this mean?](https://track.editorialmanager.com/CL0/https:%2F%2Fjournals.plos.org%2Fplosone%2Fs%2Feditorial-and-peer-review-process%23loc-peer-review-history/1/010f019790f03fb6-aaab71c6-ed23-4f38-b631-66daf8f856be-000000/lUNl0wEFtBLH659OsLaEo4SlUS7JoZEs7PP524NCrY0=216)). If published, this will include your full peer review and any attached files.


If you choose “no”, your identity will remain anonymous but your review may still be made public.


**Do you want your identity to be public for this peer review?** For information about this choice, including consent withdrawal, please see our [Privacy Policy](https://track.editorialmanager.com/CL0/https:%2F%2Fwww.plos.org%2Fprivacy-policy/1/010f019790f03fb6-aaab71c6-ed23-4f38-b631-66daf8f856be-000000/2pdZE8GpzEGGks3Av7LuqRmNTPC-Q0OprvF_cKjCgJw=216).

Reviewer #1: No

Reviewer #2: No

[NOTE: If reviewer comments were submitted as an attachment file, they will be attached to this email and accessible via the submission site. Please log into your account, locate the manuscript record, and check for the action link "View Attachments". If this link does not appear, there are no attachment files.]

While revising your submission, please upload your figure files to the Preflight Analysis and Conversion Engine (PACE) digital diagnostic tool, [https://pacev2.apexcovantage.com/](https://track.editorialmanager.com/CL0/https:%2F%2Fpacev2.apexcovantage.com%2F/1/010f019790f03fb6-aaab71c6-ed23-4f38-b631-66daf8f856be-000000/5WMkB2q3dxVgGNEsRZ_aZDOssyDe-OwaCT-PLwAQeyo=216). PACE helps ensure that figures meet PLOS requirements. To use PACE, you must first register as a user. Registration is free. Then, login and navigate to the UPLOAD tab, where you will find detailed instructions on how to use the tool. If you encounter any issues or have any questions when using PACE, please email PLOS at [figures@plos.org](mailto:figures@plos.org). Please note that Supporting Information files do not need this step.
