# Supplementary material for: IL-18 Binding Protein, a biomarker of strength maintenance after surgery but reduced physical performance in age-related sarcopenia
Source: PLoS One. 2026 Jan 27;21(1):e0340493. doi: 10.1371/journal.pone.0340493 (PMC12843554; doi:10.1371/journal.pone.0340493)
Supplement: Attachment — Submitted filename: Responses to reviewers v2.docx [file pone.0340493.s006.docx]

Dear Dr Thiyagarajan,

Thank you for the chance to re-revise the paper and I hope that the comments are now all covered. As part of the review, I have added 2 references one is a reference to the methods we used for RNA extraction (ref 28) and the other is to cover the ability of AMPK to activate PGC1-alpha and thereby promote mitochondriogenesis (ref 44). I mention this as per the instructions from the editorial office. For the remainder of the rebuttal, the editor/reviewer comments are in red, the response is in black and the alterations to the manuscript are in blue.

I hope that the paper is now acceptable for publication.

Best wishes

Paul Kemp

Response to editor’s and Reviewers’ comments:
I appreciate your responses and revision, and I agree with your concerns concerning the restriction of the NCBI submission of your data for the moment with the paper. But I would recommend submitting your data to the public domain, like NCBI, with your future work. As you have evaluated the negative enrichment of mitochondrial genes and provided a supplementary file (please mention the supplementary file number wherever it is not mentioned), it would be interesting to discuss some of the negatively enriched genes of mitochondria. I would add the same concerns to proteins as well. One of the reviewers also provided the comments and suggestions with a minor revision. Thus, I recommend you revise your manuscript.

Thank you for agreeing that we don’t need to include the submission number for our NCBI data in this paper. However, we would like to reassure you that the data have already been submitted to NCBI (as part of the process of submitting the other paper and I have an appropriate submission number). We have included reference to the additional file where appropriate.

We have also added a comment regarding the associations between specific genes that encode mitochondrial proteins and IL-18BP to the discussion. This section reads:

Consistent with increased IL-18BP associating with reduced mitochondrial content, we found that IL-18BP in plasma was inversely associated with gene sets associated with mitochondria and mitochondrial function. The genes showing these associations included components of the electron transport chain (including NDUFA4, SDHAF3, UQCRB and COX20; components of complex I, II, III and IV respectively), genes required for the synthesis of the electron transport chain (including COQ3; required for coenzyme Q synthesis) as well as genes involved in control of mitochondrially encoded RNA processing (TRMT10C).

Given that there was no original reviewer 3 we assume that the comments are from a further reviewer due to the lack of response from one of our original reviewers. We thank them for reviewing the paper and respond to their comments below. We note that we have successfully answered reviewer 1’s comments in our rebuttal and thank them for re-reviewing the work.

Response to the comments by reviewer 3.

 IL-18 Binding Protein (IL-18BP) is increasingly recognized as a context-sensitive immunomodulatory biomarker with emerging roles in muscle physiology and aging. While the current study provides valuable insights, several methodological and interpretative considerations should be addressed to enhance the manuscript’s clarity and rigor:

Study Limitations: A thorough discussion of the study’s limitations—including sample size, extract standardization, and potential confounding variables—is essential to appropriately contextualize the findings.

The section marked Study Limitations commented on the limitation of study size but predominantly with reference to the number of samples that had overlapping protein and RNA sequencing data. We have amended this to cover the study as a whole.

This section now reads

A second limitation is the sample size with the surgery cohort limited to 30 SOMAscan assay and the sarcopenia study limited to 129 individuals for the ELISA analysis. The number for which we had both transcriptomic and proteomic data was particularly limited making it possible that the associations with mitochondrial gene expression are serendipitous.

We are not clear on which aspects of “extract standardisation” the reviewer is referring to. The ELISA was performed on diluted plasma so there was no “extraction” and all samples were diluted to the same extent, the SOMAscan data was generated performed at SOMAlogic using their proprietary methods and all samples passed their QC methods.

RNA was extracted by a normal Trizol extraction method (this has now been added to the methods) and the RNA samples and libraries were QC’d at Barts Genome centre and passed their QC metrics. This has now been made explicit and the section reads

RNA was extracted from frozen muscle biopsies taken at the time of surgery (after anaesthetisation but before surgery) using TRizol as previously described [28]. RNAseq was performed at the Genome Centre, Queen Mary University of London and all samples and generated libraries passed the QC metrics used by the Genome Centre.

Data Interpretation: The observed discrepancy in IL-18BP levels between sarcopenic patients at baseline and post-surgical time points warrants further clarification. The authors are encouraged to propose specific recommendations and outline a roadmap for resolving this inconsistency.

We are unclear what discrepancy/inconsistency in IL18-BP levels the reviewer is referring to. The sarcopenic individuals (from the LACE study) did not undergo surgery. The range of IL18-BP (median 8.48pg/mL (5.76, 12.43) seen in these individuals covers the same range covered by pre-surgical levels in the patients undergoing aortic surgery (median=10.4 (8.78, 11.70). It is true that these are statistically significantly different with the surgery patients having a higher level than the LACE patients (p=0.027) though this doesn’t appear to be the difference that the reviewer is asking about as they specify post-surgery. However, given that there is a marked increase in IL-18BP following surgery compared with pre-surgery in the same individuals and we know that the surgery initiates a marked inflammatory response, and the aortic surgery patients have a significant illness associated with increased with inflammation, it seems likely that this is the most likely cause of the difference. This interpretation would also be consistent with increases in IL-18BP in sepsis where it limits the effects of IL-18 as described in the text. Other differences in the cohort include age which may also be significant but more speculative in nature as an explanation.

The difference in IL1-8BP between samples taken, before and after surgery, from the individuals undergoing surgery is, as far as we can tell, real. The samples were taken in the same way (just 24h apart from each individual over the period of about 1 year to obtain samples for the whole cohort). There were stored together at -80°C until analysed by SOMAscan and subsequently by ELISA on aliquots that were stored separately from those that went for SOMAscan analysis. In response to surgery there is a large inflammatory response with increases in many inflammatory cytokines indicating that the increase in IL-18BP is likely a consequence of this inflammatory stimulus.

Taking this together, we do not see any discrepancy in the levels of IL-18BP that could be analysed in any “roadmap”.

If on the other hand the reviewer is talking about the discrepancy in the association between IL-18BP and strength, then that is discussed in the text in the light of the effects in MACS and the mitochondrial changes in IL-18BP^-/-^ mice. However, we have added the following as a roadmap to understanding the situation in humans.

**Future directions**

Identification of the mechanisms leading to the different associations of IL-18BP that we identify in this study would require administration of either or both of IL-18 and IL-18BP or an anti-IL-18 antibody. Phase II clinical trials of IL-18BP have been started examining kidney transplantation, Chron’s disease and Behcet’s disease. These trials suggest that a clinical trial of IL-18BP in response to surgery would be practicable and could demonstrate preserved strength, comparison of this effect with that of an anti-IL-18 antibody would demonstrate that whether binding of IL-18 was sufficient to preserve strength in these patients. Determination of the mechanism by which IL-18BP reduces strength or mitochondrial function in humans is more complex. As described above data derived from mice are consistent with such an effect but experiments in humans would require prolonged treatment of patients with reduced exercise performance with very low dose IL-18 or an neutralising antibody to IL-18BP. Given the contribution of IL-18 to MACS and the protective role IL-18BP plays in this syndrome such an experiment would be unfeasible.

Title Accuracy: To ensure terminological precision, the manuscript title should reference IL-18 binding protein rather than IL-1BP.

We have modified the title accordingly.
